# Efficient Bayesian approach for multilocus association mapping including gene-gene interactions

**DOI:** 10.1186/1471-2105-11-443

**Published:** 2010-09-02

**Authors:** Pekka Marttinen, Jukka Corander

**Affiliations:** 1 Department of Biomedical Engineering and Computational Science, FI-02015 Helsinki University of Technology, Finland; 2 Department of Mathematics and Statistics, FI-00014 University of Helsinki, Finland; 3 Department of Mathematics, Åbo Akademi University, FI-20500, Finland

## Abstract

**Background:**

Since the introduction of large-scale genotyping methods that can be utilized in genome-wide association (GWA) studies for deciphering complex diseases, statistical genetics has been posed with a tremendous challenge of how to most appropriately analyze such data. A plethora of advanced model-based methods for genetic mapping of traits has been available for more than 10 years in animal and plant breeding. However, most such methods are computationally intractable in the context of genome-wide studies. Therefore, it is hardly surprising that GWA analyses have in practice been dominated by simple statistical tests concerned with a single marker locus at a time, while the more advanced approaches have appeared only relatively recently in the biomedical and statistical literature.

**Results:**

We introduce a novel Bayesian modeling framework for association mapping which enables the detection of multiple loci and their interactions that influence a dichotomous phenotype of interest. The method is shown to perform well in a simulation study when compared to widely used standard alternatives and its computational complexity is typically considerably smaller than that of a maximum likelihood based approach. We also discuss in detail the sensitivity of the Bayesian inferences with respect to the choice of prior distributions in the GWA context.

**Conclusions:**

Our results show that the Bayesian model averaging approach which explicitly considers gene-gene interactions may improve the detection of disease associated genetic markers in two respects: first, by providing better estimates of the locations of the causal loci; second, by reducing the number of false positives. The benefits are most apparent when the interacting genes exhibit no main effects. However, our findings also illustrate that such an approach is somewhat sensitive to the prior distribution assigned on the model structure.

## Background

Given the hugely decreased economic costs of utilizing large-scale single-nucleotide-polymorphism (SNP) genotyping to study the genetic architecture of a phenotype of interest, GWA analyses have become popular within many areas of molecular medicine. An excellent review [1] of the statistical challenges in GWA studies highlights the fact that no single approach has yet appeared which would comply to all immediate desiderata in this context, such as high power, reasonable control of spurious findings and relatively inexpensive computational effort. The plethora of different modeling and testing approaches for detecting single and multiple polymorphisms associated with a complex phenotype that has recently appeared in the literature demonstrates the urgent need for reliable and in practice applicable statistical methods in this context [2-9]. A variety of causal and graphical modeling ideas, as well as more standard regression modelling methods have been investigated in these works.

Some of the most challenging aspects of GWA analyses are how to sensibly handle the question of multiple model comparisons and how to identify influential gene-gene interactions since the number of putative model terms is astronomic and some interactions may involve polymorphisms that lack main effects, leading to reduced power with single-locus tests. From a theoretical statistical perspective it could be expected that the Bayesian approach [10] would provide satisfactory answers to these issues due to its ability to combine information over many models with varying parametric dimensionality. Advantages of the Bayesian methods in genetic association studies have been discussed in a recent review [11]. However, a primary burden is then how to specify a sensible prior probability distribution for all putative association models, which is a complex task [1]. The fully Bayesian approach has been employed in the context of GWA studies using regression and graphical models; however, the published approaches have not explicitly considered gene-gene interactions due to the computational burden [4,7].

In the current work we have aimed to address these challenges by developing an efficient Bayesian modeling approach that explicitly considers gene-gene interactions. Our work is partially inspired by the work of Marcini et al. where Bayesian single-locus association tests were developed [5]. Furthermore, we have examined in parallel the Bayesian graphical modeling approach introduced in [4] and discuss the particular sensitivity of Bayesian inferences with respect to the choice of prior distributions in the GWA context. We show how the marginal likelihood score can be analytically derived for a variety of Bayesian gene-gene interaction models, which in turn enables the use of highly efficient non-standard Monte Carlo model learning [12]. The advantages of such an approach compared to standard Markov chain Monte Carlo (MCMC) computation have been demonstrated for very high-dimensional model learning problems [12-14]. Moreover, contrasted with the maximum likelihood estimation of comparable logistic regression models as in [2], our method is considerably faster, which is of primary importance given the astronomic number of models that can be examined for a single data set.

The present article is structured as follows. In the *Methods *section we introduce our Bayesian model and learning algorithm for multilocus association mapping, provide a brief overview of alternative methods, and describe a simulation study utilizing real genome-wide SNP data as a basis for generating realistic levels of linkage and molecular variation. The results from the simulation study are presented in *Results*. In *Discussion*, we summarize the advantages and disadvantages of our approach and discuss some challenges encountered. *Conclusions *concisely summarizes the main points.

## Methods

### Bayesian multilocus association model

Consider a case-control study involving *N *individuals for which *y_i _*∈ {0, 1} denotes the phenotypic status such that *y_i _*= 1 and *y_i _*= 0 correspond to the presence and absence of a disease, respectively, for *i *= 1, ..., *N*. Let *Z_ij _*∈ {0, 1, 2}, *i *= 1, ..., *N *denote the observed genotype of individual *i *at SNP locus *j, j *= 1, ..., *L *Furthermore, let *π_i _*denote the probability of individual *i *carrying the disease, i.e. the event *y_i _*= 1. To simplify the notation in the model definitions introduced below, we will occasionally omit the index *i *from the disease probabilities when there is no difference between the individuals.

In a typical GWA analysis, the loci (if any) that influence disease probabilities are unknown *a priori*, such that the number of SNPs, their locations, and the form of their main/interaction effects are all unknown quantities to be inferred from the observed genotype/phenotype data. The following five association models are utilized as the basis of our mapping method. These models have been partly motivated by the models used in the simulation study in [2]:

(1) $$M_{1}(j):\pi =p_{0}I(Z_{j}=0)+p_{1}I(Z_{j}=1)+p_{2}I(Z_{j}=2),$$

(2) $$M_{2}(j):\pi =p_{0}I(Z_{j}=0)+p_{1}I(Z_{j}\in \{1,2\}),$$

(3) $$M_{3}(j,\;k):\pi =\sum_{a=0}^{2}\sum_{b=0}^{2}p_{ab}I(Z_{j}=a\;\text{and}\;Z_{k}=b),$$

(4) $$\begin{array}{l} M_{4}(j,\;k):\pi =p_{0}I(Z_{j}=0\;\text{and}\;Z_{k}=0)+p_{1}I(Z_{j}=0\;\text{and}\;Z_{k}\in \{1,2\})\\ \;+p_{2}I(Z_{j}\in \{1,2\}\;\text{and}\;Z_{k}=0)+p_{3}I(Z_{j}\in \{1,2\}\;\text{and}\;Z_{k}\in \{1,2\}), \end{array}$$

and

(5) $$M_{5}(j,\;k):\pi =p_{0}I(Z_{j}=0\;\text{or}\;Z_{k}=0)+p_{1}I(Z_{j}\in \{1,2\}\;\text{and}\;Z_{k}\in \{1,2\}),$$

**Figure 1:**
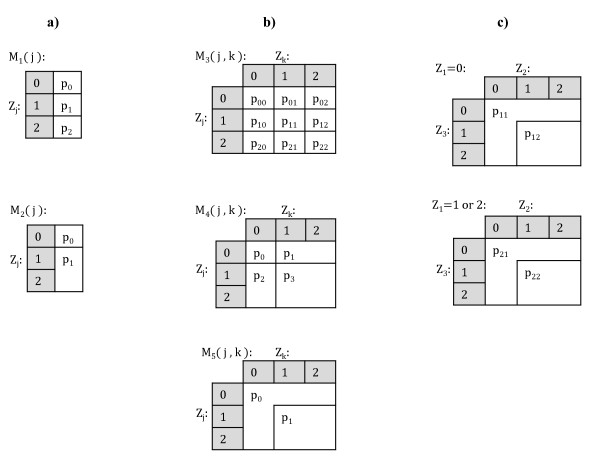
**Model specifications**. Figures 1a) and 1b) represent visually the single-locus (*M*_1_(*j*), *M*_2_(*j*)) and two-locus (*M*_3_(*j*, *k*), *M*_4_(*j*, *k*), *M*_5_(*j*, *k*)) elementary models, respectively. Figure 1c) represents visually the model obtained by combining elementary models *M*_2_(1) and *M*_5_(2, 3). This combined model is used as an example in the text.

Where *j, k *= 1, ..., *L, j *≠ *k*, and *I *(·) is the indicator function, which equals unity if the argument is true, and zero otherwise. The models specify the probability of carrying the disease given the genotype data and they are denoted by *M*_1_(·), ..., *M*_5 _(·,·), where the arguments indicate the loci involved. Interpretations of these models are given as follows. Firstly, *M*_1 _(*j*) is a full three-parameter single-locus model having the interpretation that individuals with different genotypes have different probabilities of carrying the disease. Model *M*_3 _(*j, k*) is a full nine-parameter two-locus model having the interpretation that every allele combination at loci *j *and *k *implies a different probability of carrying the disease. Model *M*_2 _(*j*) is a sub-model of *M*_1 _(*j*), corresponding to a dominant effect disease model. Models *M*_4 _(*j, k*) and *M*_5 _(*j, k*) are two-locus sub-models of *M*_3 _(*j, k*) corresponding to the dominant effect disease models with or without main effects. These five model types will be here termed as *elementary models *(see Figure 1a-b for visual representations of the elementary models). Let ${ℳ}_{e}$ denote the class of all distinct elementary models, i.e.:

$$\begin{array}{l} {ℳ}_{e}=\{M_{a}(j)|a\in \{1,2\},\;j\in \{1,\;...,\;L\}\}\int \\ \;\;\;\{M_{a}(j,\;k)|a\in \{3,4,5\},\;\{j,\;k\}\subset \{1,\;...,\;L\},\;j\neq k\}. \end{array}$$

Each elementary model specifies unambiguously a partition *S *= {*s*_1_, ..., *s_d_*} of the *N *individuals, i.e. the individuals are divided into *d *non-overlapping and non-empty subsets or classes *s*_1_, ..., *s_d _*associated with distinct disease probabilities. For instance, the elementary model *M*_1 _(*j*) specifies a partition with three classes, each corresponding to the individuals having a particular genotype at locus *j *(Z*_ij _*equals 0, 1 or 2). The following definition enables us to formally characterize our model-learning strategy.

**Definition 1**. *Let *${ℳ}_{e_{1}}\subseteq {ℳ}_{e}$*be an arbitrary subset of elementary association models. The **combined **models with respect to *${ℳ}_{e_{1}}$*are the models which can be defined by combining elementary models in *${ℳ}_{e_{1}}$*according to the following rules. (1) Select n elementary models *$M_{a_{1}}(j_{1}),...,M_{a_{n}}(j_{n})$*belonging to *${ℳ}_{e_{1}}$, *such that for any l = *1, ..., *n, a_1 _*∈ {1, ..., 5} *and either j_1 _*∈ {1, ..., *L*} *or j_1 _*∈ {1, ..., *L*}^2^, *depending on whether a_1 _*∈ {1, 2} *or a_1 _*∈ {3, 4, 5}. *(2) The locus indices j*_1_, ..., *j_n _must be disjoint, i.e. any one locus j = *1, ..., *L is allowed to be included in at most one of the models selected. (3) Let *$S_{l}=\{s_{1},...,S_{d_{l}}\}$*be the partition of individuals specified by the lth selected elementary model, and let c_1 _*= 1, ..., *d_1 _denote the index for class *$s_{c_{l}}$* of individuals in the partition S_l_, for l = *1, ..., *n. (4) Then, an association model obtained by combining *$M_{a_{1}}(j_{1}),...,M_{a_{n}}(j_{n})$*is defined by:*

$$\pi_{i}=\sum_{c_{1}=1}^{d_{1}}\cdots \sum_{c_{n}=1}^{d_{n}}p_{c_{1}\cdots c_{n}}I\left(i\in \overset{n}{\underset{l=1}{\cap }}s_{c_{l}}\right),\;i=1,\;...,\;N,$$

*where *$p_{c_{1}\cdots c_{n}}$*are arbitrary probabilities*.

As an example of the combination operator, consider the elementary models *M*_2_(1) and *M*_5_(2, 3). By omitting the index *i*, a model obtained by combining these two models is defined as:

$$\begin{array}{l} \pi =p_{11}I(Z_{1}=0\;\text{and}\;(Z_{2}=0\;\text{or}\;Z_{3}=0))+\\ p_{12}I(Z_{1}=0\;\text{and}\;Z_{2}\in \{1,2\}\;\text{and}\;Z_{3}\in \{1,2\})+\\ \;\;p_{21}I(Z_{1}\in \{1,2\}\;\text{and}\;(Z_{2}=0\;\text{or}\;Z_{3}=0))\\ p_{22}I(Z_{1}\in \{1,2\}\;\text{and}\;Z_{2}\in \{1,2\}\;\text{and}\;Z_{3}\in \{1,2\}). \end{array}$$

See Figure 1c for a visual representation of this combined model. More generally, a combined model has the interpretation that when combining two models which divide the individuals into *d*_1 _and *d*_2 _classes of unequal disease probabilities, the resulting model specifies *d*_1_*d*_2 _classes with unequal disease probabilities. These *d*_1_*d*_2 _classes correspond to all possible intersections between the classes of the two original models. Some important observations should be made about the combined models. First, the elementary models themselves, as well as the null model

$$M_{0}:\pi =p_{0},$$

are considered combined because they can be obtained through a combination involving either the elementary model itself, or no models at all. Second, the elementary models involved in a combination operation should not include any overlapping sets of loci. To demonstrate the necessity of such a restriction, consider the elementary models *M*_1_(*j*) and *M*_5_(*j*, *k*). According to the first model *M*_1_(*j*) there exists a main effect on the disease probability when the genotype at locus *j *changes. However, this contradicts the second model *M*_5_(*j*, *k*) according to which there is no such effect. The purpose of the restriction imposed on the combination operator is to prevent ambiguous model specifications in this respect. Third, the combined models are formed from main effects and two-way interactions, and do not explicitly represent higher-order interactions. For example, gene pairs AB and CD could be included, but not a triplet ABC. Nevertheless, in our approach the combination of the full models of types *M*_1 _and *M*_3 _is always permitted and it can represent even higher-order interactions; however, this comes with the expense of some redundant parameters. Indeed, the number of parameters (which is equal to the number of classes with differing disease probabilities as specified by the combined model) grows exponentially with respect to the number of loci involved in the model. The growth rate depends on whether we use full models or some lower dimensional sub-models when formulating the combined model. For example, a six-locus interaction model can have at maximum 9^3 ^= 729 and at minimum 2^3 ^= 8 parameters, depending on whether three models of type *M*_3 _or *M*_5_, respectively, are combined. Fourth, we note that the set of elementary models considered here is not exhaustive and it would be straightforward to generalize the approach by including other types of models, for example those with recessive main effects. Here our particular emphasis is on the situation where some causal SNPs lacking main effects have a joint effect, as exemplified by models of type *M*_5_. Such SNPs are expected to be most challenging to detect in practice when utilizing single SNP based statistical tests. Finally, we note that models *M*_3 _and *M*_4_, although elementary, can also be obtained by combining models of type *M*_1 _or *M*_2_, respectively. Our reason for including them as such into the pool of elementary models is to enhance the learning algorithm by making plausible two-locus combinations immediately available as building blocks for more complex models.

Next we provide some further details about the Bayesian multilocus association model. First, we derive explicitly the posterior probability *P *(*M *| *Data*) for a model, which is defined as:

(6) P(M|Data)∝P(Data|M)P(M),

where the proportionality constant does not depend on *M*. The first component of (6), the marginal likelihood, is given by

(7) $$P(Data|M)=\int \prod_{i=1}^{N}P(y_{i}|\theta )p(\theta )d\theta .$$

where *i *is the index for the genotyped individual and *θ *denotes jointly all the parameters of the association model. Let *M *be a combined model which divides the individuals into classes with unequal disease probabilities as specified by a partition *S *= {*s*_1_,..., *s_d_*}. The likelihood function is then given by

(8) $$P(y_{i}|\theta )=\sum_{c=1}^{d}p_{c}^{y_{i}}{\left(1-p_{c}\right)}^{1-y_{i}}I(i\in s_{c}),$$

where *p_c_*, *c *= 1, ..., *d*, are the model parameters. We note that the likelihood (8) is prospective, i.e. it does not condition on a possible matching of the controls to the cases (see, e.g. [15]). For example, our simulation setup is based on retrospective sampling on the basis of which we know that the numbers of cases and controls are equal. The advantage of the likelihood (8) is that it is very efficient to calculate. We use independent, symmetric prior distributions assigned on each probability *p_c_*:

(9) $$p_{c}\sim beta(\alpha ,\;\alpha ),\;c=1,\;...,\;d.$$

The values used for the *hyperparameter α *in the simulation analyses are specified in the *Specification of the search parameters *section. Together, (9) and (8) allow us to evaluate (7) analytically as:

(10) $$P(Data\;|\;M)=\prod_{c=1}^{d}\frac{\Gamma (2\alpha )}{\Gamma (2\alpha +|\;s_{c}\;|)}\prod_{b=0}^{1}\frac{\Gamma (\alpha +n_{cb})}{\Gamma (\alpha )},$$

where *n_cb _*is the number of individuals *i *in class *c *with disease status *y_i _*= *b *and |*s_c_*| is the total number of individuals in class *c*: Formula (10) specifies the standard marginal likelihood arising from the binomial likelihood under the conjugate beta distribution (see, e.g. [16]).

We assign prior probabilities on the association models using a discretized exponential distribution, specified by

(11) $$P(M)\propto \xi^{L_{M}},$$

where *L_M _*is the number of SNPs included in the model *M *and *ξ *∈ (0, 1). This prior specification implies the following properties hold:

1. If *M*_1 _and *M*_2 _are two distinct models such that *M*_2 _includes one more SNP than *M*_1_, then

$$\frac{P(M_{2})}{P(M_{1})}=\xi$$

2. The prior distribution is uniform over all combined models which include the same number of loci.

Thus, *ξ *can be interpreted as the penalty resulting from an increase in the number of SNPs in a model. In practice, it is reasonable to set *ξ *equal to a small value that depends inversely on the total number of investigated SNPs. This prevents the learning of overly complex models when the number of SNPs in a data set increases. The parameter *ξ *will be referred to as the *structure parameter *in the text. The values of *ξ *used in the analyses are specified in the *Specification of the search parameters *section.

### Stochastic search for model learning

Utilizing the elementary and combined association models, we define a Bayesian model averaging strategy for identifying evidence of disease associations among the SNP loci considered. The rationale in averaging over a set of models is that SNPs occurring often in different high-scoring models eventually get higher probabilities. For example, if a SNP is involved in interactions with many different SNPs, then the results will be averaged over models containing the alternative interactions, although all interactions are not necessarily included simultaneously in any single model. The learning process consists of the following four steps:

1. Calculate the posterior probability distribution over all elementary models in ${ℳ}_{e}$. In the previous section we derived analytical expressions for these probabilities.

2. Select a set ${ℳ}_{e_{1}}\subseteq {ℳ}_{e}$ of *K *elementary models corresponding to the *K *highest posterior probabilities, where *K *is a user-specified constant determining the accuracy of the approximate model averaging (see below). Further, include all single-locus elementary models in ${ℳ}_{e_{1}}$.

3. Run a stochastic search in the space of combined models of ${ℳ}_{e_{1}}$. For the search we use a non-reversible MCMC algorithm [12]. Search operators are described in closer detail below.

4. Let ${ℳ}^{*}$ denote the set of all combined models visited during the search algorithm in Step 3 and let ${ℳ}_{j}\subseteq {ℳ}^{*}$ denote the models in which *j*th locus is included. Let *X_j _*∈ {0, 1} denote the indicator variable of the event that locus *j *is included in the association model. The posterior probability of *X_j _*= 1 then equals:

(12) $$P(X_{j}=1|Data)=\frac{\sum_{M\in {ℳ}_{j}}P(M\;|\;Data)}{\sum_{M\in {ℳ}^{*}}P(M\;|\;Data)},$$

where *P *(*M *| *Data*) is the posterior probability of any particular association model *M*, for which the exact expression was derived in the previous section.

For the purposes of a simulation study we define a scoring scheme for loci based on the above posterior distribution (12). The approximate Bayesian model averaging (BMA) score of locus *j *is given by:

(13) $$S_{B\;M\;A}(j)=\log\left(\frac{P(X_{j}=1\;|\;Data)}{P(X_{j}=0\;|\;Data)}\right),$$

which is the logarithm of the posterior odds in favor of association.

Next we describe in detail the search operators utilized in the algorithm. Let *M *denote the current state of the search algorithm, corresponding to a combined model. Recall that a combined model is characterized by a collection of elementary models such that none of these include the same SNPs. In the following description, two elementary models are *conflicting *if they include the same SNPs. In the stochastic search algorithm we use the following three different search operators for modifying the current model.

1. Add a new randomly selected elementary model to the current model *M*. Remove from *M *all elementary models which conflict with the added elementary model.

2. Remove a randomly selected elementary model from *M*.

3. Switch an elementary model in *M *with another elementary model from ${ℳ}_{e_{1}}$. The operator simply considers jointly steps 2 and 1, in this order.

At each iteration these operators are used with the probabilities [0.5, 0.45, 0.05].

The specific feature of the non-reversible MCMC strategy developed in [12] is that the acceptance probability corresponding to a Metropolis-Hastings transition kernel is defined as

(14) $$\min\left\{1,\;\frac{P(Data\;|\;M^{*})P(M^{*})}{P(Data\;|\;M)P(M)}\right\},$$

where M* is the proposed model. Note that the ratio of proposal probabilities is omitted from the second term in (14). This defines a non-reversible Metropolis-Hastings algorithm satisfying the conditions under which it was proved in [12] that the posterior probabilities can be consistently estimated with

$$P(M\;|\;Data)=\frac{P(Data\;|\;M)P(M)}{\sum_{M\in {ℳ}^{*}}P(Data\;|\;M)P(M)},$$

where ${ℳ}^{*}$ is the set of models visited during the stochastic search. This estimation strategy is made possible by the fact that *P *(*Data *| *M*) *P *(*M*) is available analytically for all models. For a discussion of the advantages of using the non-reversible sampler in a general Bayesian model learning context, see the original article or [17], where even milder conditions for the convergence of the algorithm were provided. The stochastic search method and all alternative methods described in the next section were implemented in Matlab.

### Alternative methods for association mapping

As the first alternative scoring method for association mapping we use the p-values based on the standard log-likelihood ratio test for logistic regression models. The p-value for the *j*th locus is calculated from the log-likelihood ratio between the full three-parameter model

$$\log\left(\frac{\pi }{1-\pi }\right)=\sum_{a=0}^{2}\beta_{a}I(Z_{j}=a),$$

and the null model *M*_0_. The log-likelihood ratio statistic Λ*_j _*has asymptotically a *χ*^2 ^distribution with 2 degrees of freedom (see [18]). Thus, the marginal p-value based score is defined by:

(15) $$S_{p-value}(j)=-\log_{10}(P(\chi_{2}^{2}>\lambda_{j})).$$

The second alternative score for a locus *j *is derived by calculating p-values for interaction models (abbreviated as GxG models) between loci *j *and *k*, for all *k*, using the logistic regression model:

$$\log\left(\frac{\pi }{1-\pi }\right)=\beta_{0}+\beta_{1}I(Z_{j}>0)+\beta_{2}I(Z_{k}>0)+\beta_{3}I(Z_{j}>0\;\text{and}\;Z_{k}>0)$$

The fitted interaction models have four parameters analogously to the elementary model *M*_4_, and the corresponding log-likelihood ratio statistic Λ*_jk _*follows asymptotically a *χ*^2 ^distribution with three degrees of freedom. The interaction p-value based score is thus defined by:

(16) $$S_{GxG},{\;}_{p-value}(j)=\;\underset{k=1,...,L,k\neq j}{\max}\;\{-\log_{10}(P(\chi_{3}^{2}>\lambda_{jk}))\}.$$

A similar p-value could alternatively be calculated using the Mantel-Haenszel test (see, e.g. [15]).

The third alternative is similar to the second one in the sense that it tests marginally (in the posterior sense) the disease association for each pair of loci. However, the score is based on Bayes factors [19]:

(17) $$S_{GxG}{}_{,}{\;}_{BF}(j)=\underset{k=1,...,L,\;k\neq j}{\max}\left\{\log\left(\frac{P(Data|M(j,k))}{P(Data|M_{0})}\right)\right\},$$

where

(18) $$P(Data|M(j,\;k))=\frac{1}{3}\sum_{a=3}^{5}P(Data|M_{a}(j,\;k)),$$

where *P *(*Data *| *M_a _*(*j*, *k*)) is the marginal likelihood based on the two-locus elementary model *M_a _*(*j, k*) (see *Bayesian multilocus association model*), and *M*_0 _is the null model according to which all individuals have the same disease probability. Thus, the probability of the data under the two-locus association model (18) is given by a mixture of three interaction models of different complexities, each having a prior weight of 1/3. (As an alternative to averaging over the three models, we also considered taking the maximum over the models; however, no notable changes in the results were detected.) Notice the difference between the Bayesian scores (13) and (17). In (13) the posterior probability of disease association for locus *j *is obtained by summing the posterior probabilities of all models in which *j *is included. In (17) the posterior probability of association for locus *j *is based on a single two-locus interaction model (which, however, is a mixture of three models) maximizing the probability of data. Analytical expression for the above Bayes factor is based on the derivations provided in the section *Bayesian multilocus association model*.

### Simulated data sets

As the basis of our simulations we use data on 2131 real human subjects with approximately 500,000 SNPs from the autosomal and X chromosomes. The data belong to GenMets sample collected as part of the Health2000 study. Less than 0.1 percent of the observed SNPs were missing in the original data. For the purposes of the simulation study, we impute the missing values by drawing the missing alleles from the marginal allele distributions of the corresponding SNPs. Further details about the characteristics of the data can be found from previously published studies utilizing the data [20,21]. We carry out experiments with two types of data sets: smaller data sets consisting of 460 SNPs are used in replicate experiments to investigate the average performance under various biological scenarios, and larger whole chromosome data sets consisting of approximately 8,700 SNPs are used as examples of the performance in a computationally more challenging scenario.

The simulated data sets for the replicate experiments contain subsets of the actual genotype data and the disease status is generated for the corresponding individuals according to one of three different disease models, inspired by the simulation settings used in [2] and [4]. This mechanism of data synthesization preserves well the level of challenge related to screening disease-related loci from real data. The outline of the simulation procedure for generating a single data set is as follows:

1. We specify genotype relative risk (GRR), minor allele frequency (MAF) for the causal SNPs, and a generative model for the disease status (see below).

2. We randomly select from among the 500,000 original loci four causal SNPs from different chromosomes whose empirical allele frequencies (calculated from all 2131 individuals) match closely (∓1%) the specified MAF. For each of the selected causal SNPs, we select the genomic area surrounding the causal SNP such that 20 closest flanking SNPs having *MAF *> 0.1 on both sides of the SNP are included in the area. The restriction to SNPs with *MAF *> 0.1 reflects the ascertainment bias, and is similar to the simulations in [4]. The true causal SNP is then excluded from the data for the corresponding area, which mimics the situation where causal variants are linked to the genotyped loci but their exact locations remain hidden in a study. This procedure yields 4 genomic intervals, each of length 40 SNPs, such that the causal SNPs are located in the centers of the intervals (but not included in the observed data). These intervals represent disease associated genomic areas. We note that the linkage between the causal SNP and the neighboring SNPs is not constant in the resulting data sets; however, the results are averaged over in this respect in the simulations.

3. We randomly select three intervals of length 100 SNPs from chromosomes not harboring any of the selected causal SNPs. These intervals represent genomic areas not associated with the disease.

4. We concatenate the genomic intervals to a multilocus genotype sequence of length 460 SNPs such that the intervals from Step 3 are inserted between the disease associated intervals from Step 2.

5. Based on the four causal SNPs and the specified disease model, we generate a disease status for each of the 2131 individuals in our real genotype data using their observed genotypes. We select the values of the parameters in the disease models to get a prevalence of 40 percent (for details see below). This leads to approximately 850 observed cases in a single data set.

6. We select randomly the controls from the remaining set of appr. 1280 individuals to obtain a data set with an equal number of cases and controls. The final data consist of the genotypes of the selected cases and controls at the 460 SNPs and their simulated disease statuses according to the generating model.

We generate 100 replicates of synthetic data sets for each combination of the simulation parameters (for details see below) and use these to assess the relative performance of the association mapping methods considered. Let *j*_1_, ..., *j*_4 _denote the causal SNPs selected. The following three models are used for generating the disease statuses:

(19) $$\pi =\beta_{0}*GRR^{I(Z_{j_{1}}>0)+I(Z_{j_{2}}>0)+I(Z_{j_{3}}>0)+I(Z_{j_{4}}>0)},$$

(20) $$\pi =\beta_{0}*GRR^{I(Z_{j_{1}}>0\;\text{and}\;Z_{j_{2}}>0)}*GRR^{I(Z_{j_{3}}>0\;\text{and}\;Z_{j_{4}}>0)},$$

and

(21) $$\pi =\beta_{0}*GRR^{I(Z_{j_{1}}>0\;\text{and}\;Z_{j_{2}}=0)*Z_{j_{3}}}*GRR^{I(Z_{j_{4}}>0)/2},$$

where *β*_0 _is baseline risk chosen such that the resulting prevalence meets the value specified in Step 5 above. According to the first generative model, the risk of having the disease increases multiplicatively (i.e. additively on the log scale) by (*GRR *- 1) * 100% whenever any of the loci involved has at least one disease-related allele. According to the second model we have two pairs of interacting loci, (*j*_1_, *j*_2_) and (*j*_3_, *j*_4_), such that the risk of having the disease is increased by (*GRR *- 1) * 100% if both loci in either pair have at least one risk allele, and the risk is multiplicative across the locus pairs. The third generative model requires simultaneously that $Z_{j_{1}}>0$, $Z_{j_{2}}=0$ and $Z_{j_{3}}>0$, before the risk increases. This increase is then multiplicative with the increase caused by *j*_4 _alone. The generative models will be referred to as "multiplicative", "threshold" and "triplet", respectively.

In the simulation setup, we use values: GRR = (1.3, 1.6, 2.0) and MAF = (0.05, 0.1, 0.2) for the causal SNPs. For each of these MAF values, the original data set includes more than 23,000 SNPs to choose from. The simulation setup thus leads to 27 different parameter settings (9 for each of multiplicative, threshold and triplet generative models) and 100 replicate data sets are generated for each setting.

In addition to the 2,700 data sets of size 460 SNPs generated in the way described above, we simulate two whole chromosome data sets which include approximately 8,700 SNPs each. These data sets are generated using the threshold model with values GRR = (1.6, 2.0) and MAF = 0.2. The disease associated genomic areas and disease statuses are generated exactly as before, except that chromosome 21 is excluded as a possible origin for any of the causal SNPs. The areas not associated with the disease are created by dividing all SNPs in the 21st chromosome into five intervals of approximately the same size, and the complete data sets are obtained by inserting the disease associated genomic areas between these intervals.

### Specification of the search parameters

As the initial model for the search, we used the empty model which includes no SNPs. To fully specify the search algorithm, *K *and *N*_*iter *_must be set, where *K *is the number of elementary models whose combinations define the search space and *N*_*iter *_is the number of iterations in the stochastic search algorithm. These parameters were specified and the convergence of the search was monitored in the different simulations as follows:

• In the analyses of the data sets with 460 SNPs we used *K *= 5, 000 and *N_iter _*= 200, 000. The convergence of the search algorithm is investigated by manually inspecting the marginal likelihood trace plots for approximately ten different data sets and the convergence was always reached within the first 20,000 iterations. The same values were used in the search for all the data sets.

• In the analyses of the two whole chromosome data sets we used *K *= 50, 000 and *N_iter _*= 3, 500, 000. The convergence of the search was investigated by manually inspecting the trace plot after each 500, 000 iterations. For both data sets, the convergence was reached during the first set of 500,000 iterations. Further, the highest scoring model did not change after the first 500, 000 iterations in either of the analyses.

Although the likelihood (8) is based on a prospective model (see the section *Bayesian multilocus association model*), we can utilize the prior knowledge that the numbers of cases and controls in a data set are equal by selecting the hyperparameter *α *in the distribution (9) to reflect this information. This hyperparameter specifies the distribution of the disease probability parameters, and specifically, by selecting *α *> 1 we give more weight to disease probabilities close to 0.5. In the supplementary material of their article, Marchini et al. interpret the hyperparameter in terms of odds-ratios, and conclude that hyperparameter values larger than unity are better in line with odds-ratios one would expect from typical diseases than values less than unity [5]. If not stated otherwise, we used *α *equal to 3 in our analyses. However, we note that this choice is still fairly non-informative, and the results obtained were practically identical when *α *= 1 was used (see the next section). Furthermore, in the replicate simulations we used the value 1/460 for the structure parameter *ξ *in (11). In the investigation of the sensitivity of the inferences with respect to the priors, we considered also values *α *= 1, 3, 10 and *ξ *= 1/46, 1/460, 1/4600. In the whole chromosome analyses we used *ξ *= 1/1000.

## Results

### Simulations

**Figure 2:**
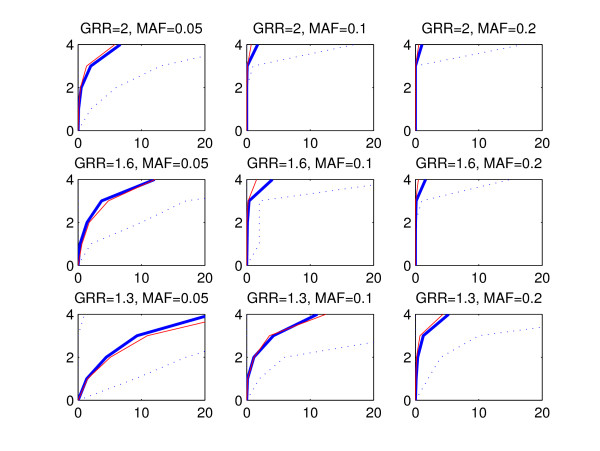
**Mean ROC curves, multiplicative model**. The figure shows the average ROC curve (thick blue line) together with 2.5th and 97.5th percentiles (dotted lines) for the BMA method (see the text for further explanation). For comparison, the average ROC curve corresponding to the p-value method is shown (red line). The horizontal axis corresponds to the average count of false positives and the vertical axis shows the number of detected disease associated areas. The plot in each panel is based on 100 simulated data sets. The data sets were generated according to the multiplicative model, and the values of GRR and MAF parameters are shown on top of the respective panels. Notice that the scale of the horizontal axis in this figure is different from the scales in Figures 3 and 4.

**Figure 3:**
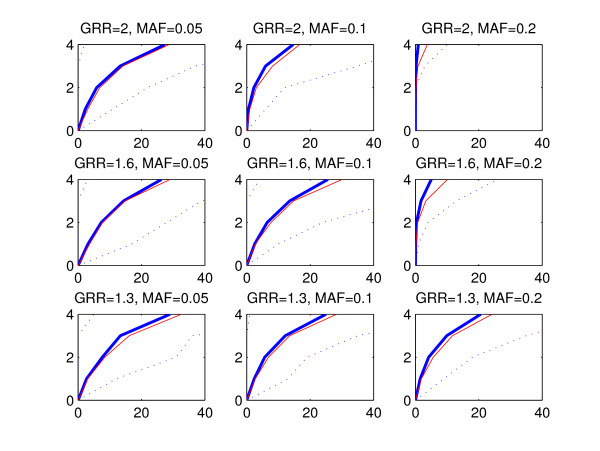
**Mean ROC curves, threshold model**. The figure is interpreted similarly to Figure 2, except that the data sets were generated according to the threshold model.

**Figure 4:**
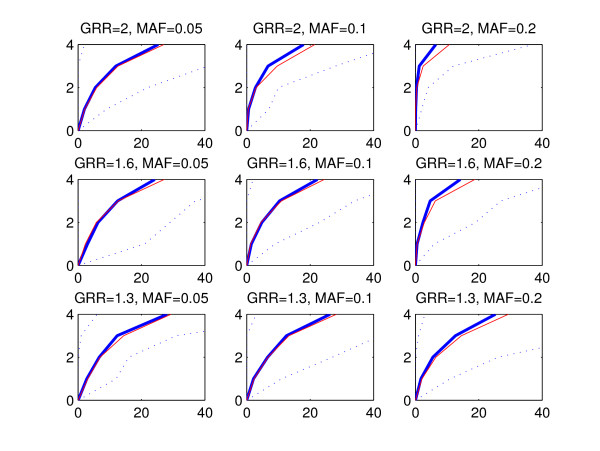
**Mean ROC curves, triplet model**. The figure is interpreted similarly to Figure 2, except that the data sets were generated according to the triplet model.

The statistical performance of a method can be evaluated by considering a subset of highest ranking SNPs and investigating how many disease associated areas are detected by these SNPs and how many false positive findings, i.e. SNPs from outside of the disease associated areas, are included in the subset. To refrain from fixing the size of the subset, we utilize a graphical representation where the number of disease associated areas detected is plotted against the count of false positive SNPs. We call these curves ROC curves due to the apparent similarity with the commonly used receiver operating characteristic (ROC) curves [22], where the true positive rate is plotted against the false positive rate. However, in our presentation the true positive rate is replaced by the number of disease associated areas detected, because we do not expect that all SNPs within a single disease associated area would be assigned high scores. Figures 2, 3 and 4 show the ROC curves for the BMA (blue) and p-value (red) methods in the replicate simulations when the data sets are generated using multiplicative, threshold or triplet models, respectively. The curves are averaged by calculating the mean count of false positive SNPs in the 100 replicate data sets for each number of disease associated areas detected. The vertical axis corresponds to the number of detected disease associated areas and the mean count of false positives is shown on the horizontal axis. Variability over data sets is displayed by the 2.5th and 97.5th percentile curves (dotted blue) for the BMA method. These percentiles represent the tails of the distribution of counts of false positives evaluated at each number of disease associated genomic areas detected. When interpreting the results, the red curve for the threshold model with GRR = 1.3 and MAF = 0.05 (Figure 3, lower left-hand panel) can be used as a baseline showing the highest relative false positive rate. When we decreased the effect size further from 1.3 to 1, the resulting curve for the p-value was nearly identical with the curve shown in the figure (exact results not shown). Note that this baseline curve is not a straight line, because, unlike in standard ROC curves, the vertical axis does not here represent the number of true positive SNPs, but the number of true positive disease associated areas detected. The following conclusions can be drawn from the figures:

• Detection of causal areas improves with increasing GRR and MAF. Especially under the threshold and triplet simulations, if the conditions *GRR *≥ 1.6 and *MAF *≥ 0.1 are not satisfied, no method is clearly better than the baseline.

• There is considerable variability between the curves for different data sets, as can be seen from the wide 95% intervals for the curves. This is expected as the data sets are based on subsets of real genotype data and may exhibit different levels of linkage between the causal and neighboring SNPs.

• The performance of the methods is highest with multiplicative data sets and lowest with triplet data sets.

• The mean curve for the BMA is consistently above the p-value curve in triplet and threshold simulations. In multiplicative simulations the curve for the p-value is above the curve for the BMA, except when MAF = 0.05 and GRR = 1.3 or 1.6. We note that when the signal gets stronger, even the marginal p-value is able to identify interacting SNPs without main effects, because such SNPs show some effect also marginally when averaged over the other SNP.

For clarity, the ROC curves for the GxG p-value and GxG BF methods are excluded from Figures 2, 3 and 4. Usually these curves reside between the p-value and BMA curves (exact results not shown).

**Figure 5:**
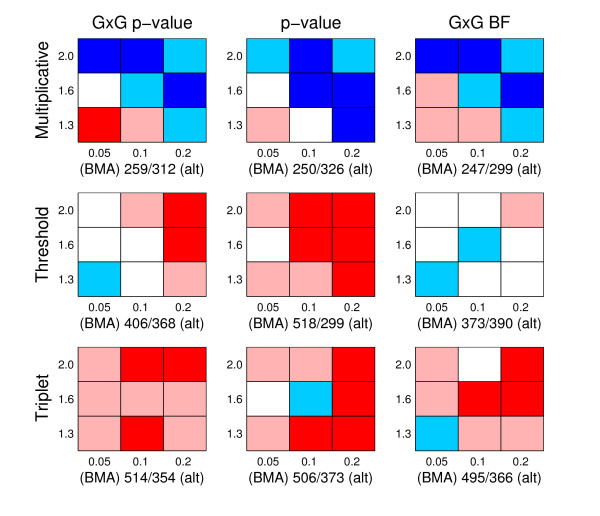
**Comparison of AUC**. The figure summarizes pairwise comparisons based on AUC values between BMA and alternative methods. There are nine panels in total, corresponding to all possible pairs of generating model (multiplicative, threshold, triplet) and alternative method (GxG p-value, p-value and GxG BF). The rows of panels correspond to different generating models, as specified on the left side of the rows, the columns of panels correspond to comparisons with different alternative methods, which have been specified on top of the columns. Each panel is divided into nine cells, and each cell summarizes results from one hundred simulated data sets. MAF values 0.05, 0.1 and 0.2 are in columns 1-3, respectively, whereas GRR values 1.3, 1.6 and 2.0 correspond to the rows 1-3 (from bottom to top), respectively. For each cell, we calculated the number of times AUC value was higher/lower for BMA than for the alternative method among 100 simulated data sets, whenever the methods had different values. A cell is colored to reflect the result of this comparison, and the colors are interpreted as follows: Red: BMA achieves a higher AUC value more often than the alternative method and the difference is statistically significant. Light red: BMA achieves a higher AUC value more often, but the difference is not statistically significant. White: the difference between the number of data sets in which BMA gets a higher score and in which BMA gets a lower score is less than 5, meaning that there is in practice no difference between the methods. Light blue: BMA achieves a higher score less often than the alternative method, but the difference is not statistically significant. Blue: BMA achieves a higher score less often than the alternative method, and the difference is statistically significant. The statistical significance of the difference is measured using a two-tailed p-value based on a binomial distribution using significance level of 0.05. Below each panel we show two values *a/b*, where *a *is the number of times BMA gets a higher AUC score, and *b *the number of times BMA gets a lower score than the alternative method over all the nine cells.

**Figure 6:**
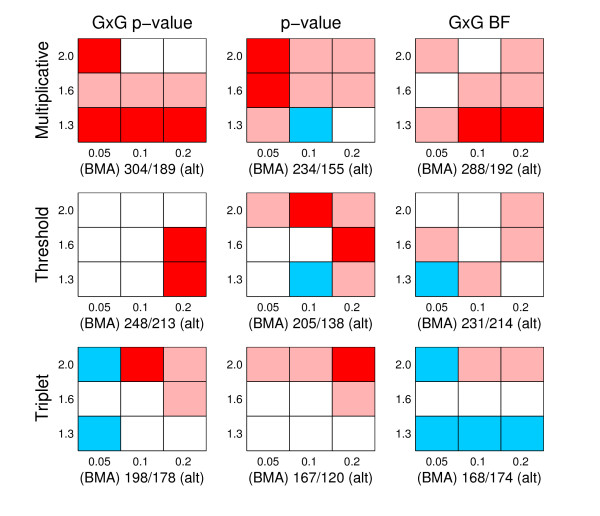
**Comparison of location accuracy**. The figure summarizes pairwise comparisons of location accuracy between BMA and alternative methods. The figure should be interpreted analogously to Figure 5, except that the comparison is based on location accuracy instead of AUC values (preference for BMA is still indicated using the red color).

To numerically compare the alternative methods, we normalize the axes in our ROC curves to unity and calculate the area under the ROC curve (AUC). In general, the higher the AUC value, the better a method is performing in the identification of the disease associated genomic regions. We performed a pairwise comparison of the results for each data set created in the replicate simulations between BMA and the alternative methods (p-value, GxG p-value and GxG BF). Two different criteria are used: first, the AUC value; second, the location accuracy. The location accuracy is defined by taking the highest ranking SNP and measuring its distance in terms of SNP markers to the nearest causal position. If the highest ranking SNP resides outside of any causal area the distance is set to be the maximum possible value (which equals 20, since all disease associated areas had length 40 and the true causal SNPs were located between the 20th and 21st SNPs). However, in the triplet simulations we modify the location accuracy criterion so that we only consider SNPs outside the causal area related to the causal SNP $Z_{j_{4}}$ in Equation (21). The reason for this is that the SNP $Z_{j_{4}}$ is the only SNP in the triplet model (21) which has a main effect, and for this reason it is usually easiest to detect and gets the highest rank. In the multiplicative simulations the location accuracy is already investigated using causal SNPs with main effects, therefore it would be unnecessary to repeat this with the triplet simulations. The results of the comparisons are jointly presented in Figures 5 and 6, and they reveal that:

• When AUC is considered, BMA is significantly better than any other method in triplet simulations, and, in threshold simulations, only the GxG BF method is competitive with the BMA method. On the other hand, in multiplicative simulations, especially when the signal is strongest (upper right corner), BMA gets lower AUC values than the alternatives.

• When the location accuracy is considered, BMA has consistently better or approximately equal performance compared to the alternatives. In multiplicative data sets the preference for the BMA is strongest.

The most striking feature in the results is the fact that the BMA method is clearly inferior in terms of AUC when the data sets are generated according to the multiplicative model and the signal is strongest, and, at the same time, clearly superior to the other methods with multiplicative data sets when measured in terms of location accuracy. The relatively low performance of BMA in terms of AUC for this setting can be explained as follows. When the signal was strongest and the risk increased multiplicatively over the causal loci, it happened with some few data sets that BMA did not show any signal to one of the four causal loci. Consequently, many false positives needed to be included before all four disease associated areas could be appropriately detected. This is also visible in Figure 2, where, for example, in the panel corresponding to GRR = 2.0 and MAF = 0.2 the mean ROC curves for BMA and p-value are overlapping up to three detected causal areas, but the 97.5th percentile deviates strongly from the mean curves at the level of four detected disease associated regions. The reason why models with four causal SNPs included sometimes get lower posterior probabilities than models with three causal SNPs can be explained by considering the generating multiplicative model. When the increase in risk is maximal, the risk is already considerably high after including any three SNPs in the model, and, consequently, only a minor increase in risk is left to be explained by the fourth causal SNP. Thus, the benefit from adding this particular SNP to the model will not always compensate the penalty resulting from the corresponding increase in the number of parameters. On the other hand, the location accuracy criterion only compares the location accuracy of the highest ranking SNP in the data set, and is therefore unaffected by this phenomenon.

As a final illustration of the results from the replicate simulations, Table 1 shows summary information about the variation in posterior odds values that was observed in the replicate simulations for SNPs in disease associated areas with different levels of MAF and GRR. These results confirm the expectation that the larger the effect, the higher the scores related to the disease associated areas. The table also shows the proportion of data sets in which some SNP from a disease associated area was assigned the highest score among all SNPs in a data set. Because the disease associated areas cover 160/460 ≈ 0.35 of the sequence in these data sets, the baseline proportion is about equal to 0.35. The results confirm that when GRR = 1.3 or 1.6 and MAF = 0.05 or 0.1 in the threshold simulations, or GRR = 1.3 and MAF = 0.05 in the triplet simulations, the improvement in the detection of disease associated areas provided by the BMA method is negligible.

**Table 1:** SNP posterior odds summaries

Type	GRR	MAF	Max Score	Accuracy
Multiplicative	1.3	0.05	[-6.1,1.6]	0.57
	1.3	0.1	[-4.6,7.0]	0.89
	1.3	0.2	[-2.0,11.3]	0.96
	1.6	0.05	[-6.0,6.8]	0.88
	1.6	0.1	[-0.5,44.9]	0.96
	1.6	0.2	[0.6,71.9]	1.00
	2.0	0.05	[-0.6,23.9]	0.95
	2.0	0.1	[1.2,77.6]	1.00
	2.0	0.2	[3.8,129.4]	0.99

Threshold	1.3	0.05	[-6.8,-1.0]	0.38
	1.3	0.1	[-6.4,-0.6]	0.34
	1.3	0.2	[-6.2,0.2]	0.55
	1.6	0.05	[-7.0,-1.3]	0.34
	1.6	0.1	[-7.4,0.3]	0.38
	1.6	0.2	[-3.4,16.3]	0.96
	2.0	0.05	[-6.3,-0.6]	0.40
	2.0	0.1	[-6.1,8.1]	0.80
	2.0	0.2	[-0.7,43.5]	1.00

Triplet	1.3	0.05	[-6.2,-1.3]	0.28
	1.3	0.1	[-6.3,1.1]	0.41
	1.3	0.2	[-7.7,0.5]	0.47
	1.6	0.05	[-6.9,0.6]	0.41
	1.6	0.1	[-9.9,0.6]	0.59
	1.6	0.2	[-5.4,4.6]	0.85
	2.0	0.05	[-7.2,1.6]	0.43
	2.0	0.1	[-5.5,11.7]	0.80
	2.0	0.2	[-2.7,22.8]	0.95

The table shows summary information about the maximum BMA scores, i.e. log posterior odds values, obtained for SNPs residing in the causal areas in the simulated data sets. The Max Score column reports the empirical 95% interval for the highest score observed for a SNP within any causal area in a data set. The Accuracy column reports the proportion of all data sets in which the SNP assigned the highest score among all SNPs in a data set belonged to a causal area. The values reported on each row are based on 100 simulated data sets.

**Figure 7:**
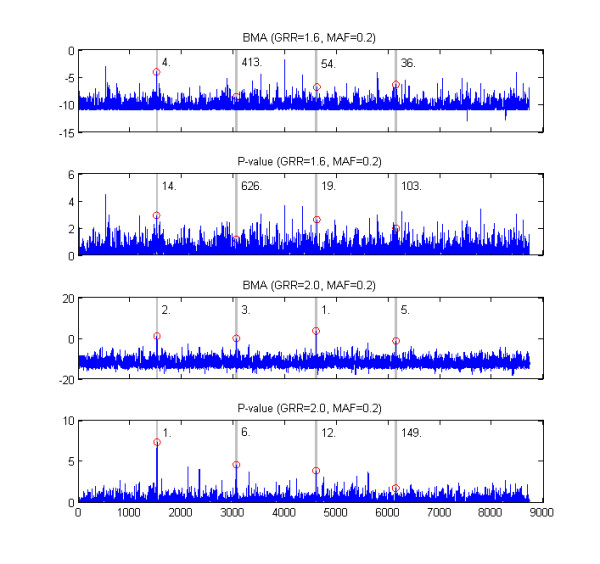
**Whole chromosome examples**. The figure shows results for the two simulated whole chromosome data sets. The two panels on top show BMA and p-value scores for each sequence position in the first data set, the lowest two plots show corresponding results for the second data set. The numbering of the sequence positions is the same in all panels, and it is shown only below the lowest panel. The four disease associated areas are highlighted using grey backgound. The highest score within each disease associated area is marked by a red circle and its ranking among all SNPs is shown next to the disease associated area. The data sets were generated using the threshold model and the values of the parameters used (GRR and MAF) are shown above the panels.

The results for the whole chromosome analyses are shown in Figure 7. These results illustrate in particular what benefits BMA based approach can provide over p-values when applied to larger genomic segments. For both data sets analyzed, the BMA method gives on average higher rankings to SNPs from disease associated regions. The benefit is clear especially when GRR = 2.0 and MAF = 0.2, when the BMA is able to identify all four disease associated areas whereas p-value misses one disease associated area completely.

### Time intensity of the methods

Obviously, calculation of the marginal p-values is the optimal approach in terms of time intensity as the time required is linear with the number of SNPs *L *in a data set, whereas going through all gene pairs for obtaining the GxG p-values takes a time proportional to $\left({}_{2}^{L}\right)$. The calculation of GxG BF scores takes approximately one third of the time required for the calculation of GxG p-value scores and this ratio does not depend on the characteristics of simulated data sets. Most of the calculation time for GxG p-values was consumed by the fitting of the logistic regression model, for which purpose we used *glmfit *from the Matlab statistics toolbox. The numerical fitting takes considerably longer time than the analytical evaluation of the GxG Bayes factors. Notice also that GxG Bayes factors are based on the average of three models as opposed to the single model in the GxG p-value. The time consumed by the stochastic search algorithm for BMA depends on the number of elementary models *K*, whose combinations define the search space, and the number of iterations of the search algorithm. In our analyses of the chromosome-wide data the calculation of all GxG Bayes factors took about 12 hours on a single desktop computer, while the stochastic search required only about 15 minutes when GRR = 1.6 and 50 minutes when GRR = 2.0. This difference in the times is a consequence of the fact that when the signal is strong the algorithm visits higher-order models more often and the evaluations of such models take longer.

### Sensitivity to prior

**Figure 8:**
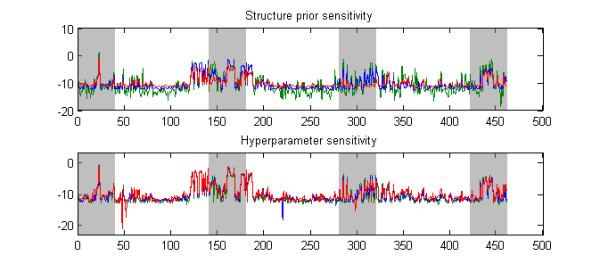
**Prior sensitivity illustration**. The figure shows the effect of varying the priors when calculating BMA scores for a single data set. In the upper panel, values 1/46, 1/460, 1/4600 (green, blue, red) were used for the structure prior parameter *ξ *with a fixed hyperparameter *α *equal to 3. In the lower panel, hyperparameter values 1, 3, 10 (green, blue, red) were used for *α *in the analysis with the structure prior parameter *ξ *fixed to 1/460.

The sensitivity of the BMA score, i.e. the logarithm of the posterior odds, to different choices of prior parameters is illustrated in Figure 8, which shows the results for a randomly selected threshold data set with GRR = 1.6 and MAF = 0.2 using three alternative hyperparameter or structure parameter values. By examining Figure 8, it is obvious that the structure prior parameter *ξ *has a considerable effect on the calculated BMA scores. In particular, making *ξ *larger increases the variance of the scores. On the other hand, the hyperparameter has only a small impact on the results, as the curves in the second plot in Figure 8 are very closely overlapping. Notice that few downward "spikes" in scores in the lower plot were not caused by different prior parameters but by the fact that the search algorithm failed in some analyses to visit a low-scoring model visited in other analyses, and, consequently, the posterior odds for the corresponding SNP were exceptionally low. Such problems could be circumvented by running a longer search. However, as these downward spikes were always observed in SNPs that would nevertheless be assigned low scores in the end, we do not consider this to represent a serious issue. Of course, there is no absolute guarantee that such events would not happen for SNPs that should in reality get high scores, unless the search is run infinitely long. In practice this is still unlikely to happen, because the search is directed toward models with high scores.

**Figure 9:**
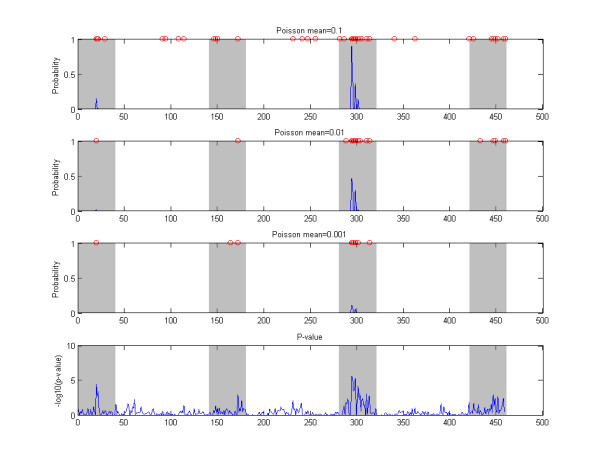
**Example, Bayesian graphical models**. The top three panels show marginal probabilities of association obtained by a Bayesian graphical model analysis (see text). The results are shown for a single example data set generated according to the threshold model with GRR = 1.6 and MAF = 0.2. The results are obtained by using three different mean paramater values for the Poisson prior distribution and the values used are shown in the labels of the panels. The SNPs with non-zero probabilities are further highlighted by red circles on top of each of the panels. Disease associated genomic areas are indicated with gray background. The lowest panel shows the marginal p-values for reference.

The prior on the model structure affects the calculated model averaged results in two ways. First, the prior probabilities of the models over which the averaging is perfomed change as a function of the structural prior. Second, the set of models over which the averaging is done can change as well, because the MCMC algorithm will eventually traverse a subspace of different models. A major challenge in specifying reasonable priors is that there is a large difference in the numbers of models of different dimensions. For example, a single SNP can be selected to the model in *L *ways, where *L *is the total number of SNPs, two SNPs can be selected in $\left({}_{2}^{L}\right)$ ways etc. If we specify a reasonable prior on the number of SNPs, e.g. a binomial distribution with some mean *µ*, then adding a SNP to the association model decreases the prior probability approximately by a factor equal to *µ*/L: When *L *becomes large the factor diminishes. Thus, although the models of higher dimension might together affect the posterior probabilities significantly, any single model of higher dimension is *per se *so improbable that an MCMC algorithm will only seldom accept a visit to such a model and consequently the search process will visit only a fraction of the putative higher dimensional models. The sensitivity of the Bayesian model averaging to the prior probabilities on model structure seems not to be specifically related to our formulation of the association model. For example, [4] used Bayesian graphical models to identify disease associations. We have implemented the approach described in their article, except that the posterior probabilities are calculated by using (12), instead. Figure 9 shows the results from that alternative method for a randomly selected synthetic threshold data set. The data are analyzed with three different Poisson priors on the number of disease associated components in the graph considered, corresponding to the mean parameters 0.1, 0.01, 0.001, where 0.01 is the value used in [4]. However, their prior definition leaves some room for interpretation. Namely, it is not explicitly stated how the prior probability mass is distributed over the different models having the same number of disease associated components in the graph. Thus, the Poisson prior can be considered to imply either that the Poisson probability mass *p*(*K*) is evenly distributed among all models with *K *disease associated components, or that all possible models in the considered model space have a prior probability directly proportional to *p*(*K*). The former interpretation leads to an extremely conservative prior, which is unlikely to lead to the detection of any disease associations, and thus, we chose the latter interpretation in our implementation. Notice that although the Poisson prior with mean 0.01 seems conservative under the latter interpretation, as the model with no associations is approximately 100-fold more probable than any particular model with a single association, the total prior probability given to models with associations is still large compared to the probability of no association, since the number of models with at least one or more associations is very large. These observations illustrate well the challenge related to the choice of a sensible prior in the current context, as one needs to balance between the two extremes of assigning too little or too much prior belief in the existence of associations.

## Discussion

### Rationale of the multilocus modeling

**Figure 10:**
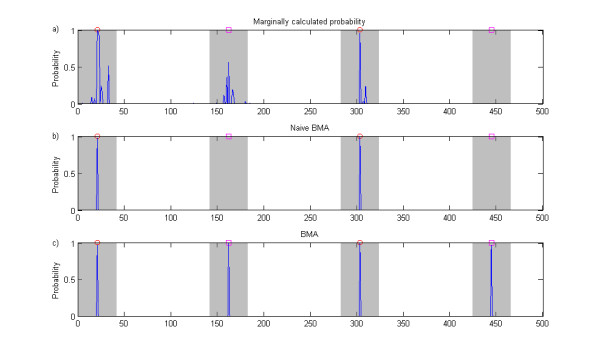
**Naive Bayesian model averaging illustration**. The figure shows association probabilities for SNPs in a single data set obtained using three different approaches. The data set is generated according to the threshold model, i.e. it includes two pairs of interacting SNPs. However, for purposes of illustration, this data set is simpler than those analyzed in the simulation experiments as the causal SNPs are included in this data set. Furthermore, the relative risks for the different interactions were selected unequal: the interaction involving SNPs denoted by red circles has relative risk 2.0 and the one involving SNPs denoted by magenta squares has relative risk 1.8. The probabilities in panel a) are calculated by comparing a single-SNP association model with the null model for each SNP in turn. Panel b) shows the probabilities from naive model averaging, where the averaging is done over all elementary single-SNP and GxG models selected for the analysis, but not including combined models. Panel c) shows the probabilities obtained from the full BMA analysis.

A strong rationale behind the models which involve multiple SNPs simultaneously is that only the SNPs providing additional information about disease risk over the SNPs already included in the model have a non-negligible chance of becoming eventually added to the model. Therefore, fewer SNPs corresponding to the strongest signals per disease associated genomic area attain high scores when the models are averaged over (for an illustration, compare panels a and c in Figure 10). From the theoretical point of view, the expected benefits include the lower number of false positives, improved power, and improved localization of the causal SNPs. In the GWA setting, these advantages have been illustrated in practice using a multilocus regression model [7], however, without including the gene-gene interactions.

Our primary target was to develop a model-averaging approach in which gene-gene interactions are explicitly considered. Another possibility for implementing this would be to consider enumeratively all single-SNP and GxG models as in our approach, while doing the model averaging over all these models. We considered this alternative at an initial stage of our method development; however, such an approach was discovered to generally suffer from a specific deficiency. To illustrate this, suppose for example that there exist two separate underlying interactions for a particular data set, i.e. corresponding to four causal SNPs in total. Then it is likely that the individual GxG models for each of these interactions are assigned high scores relative to the null model. Nevertheless, due to stochasticity of the genotype counts, it may also easily happen that one of the GxG models is assigned a clearly higher score than the other. Because this model includes only one of the interactions, and excludes the other, evidence against the other interaction is obtained. As a consequence of this, the interaction associated with the lower score will get a very low posterior weight when the averaging over all models is performed (for an illustration, compare panels b and c in Figure 10). To resolve this issue, it is necessary to have the ability to include both interactions simultaneously in a model. This insight from the initial investigations led us to propose our final approach based on the combined models. An additional benefit of the combined models is that they can represent even higher-order interactions, however, this comes with the expense of an increase in the number of redundant parameters, which is likely to reduce the applicability when the number of SNPs involved in the combination is large.

### Summary of the results

To compare our novel approach with some standard approaches we carried out a comprehensive simulation study. The simulation study was particularly challenging as the causal variants were not included among observations in the simulated data sets. In the simulations, three types of causal SNPs were considered, 1) SNPs with main effects, 2) SNPs without main effects but with a pairwise interaction effect, and 3) SNPs included in a three-way interaction without pairwise effects. The results show that even if multilocus association findings may lack statistical significance under stringent criteria for the posterior odds score, the calculated relative scores still often correctly highlight the disease associated genomic areas.

The results concerning the detection rate versus false positive rate can be summarized as follows.

• When the causal SNPs had main effects, the BMA did not provide improvement over the other methods. On the contrary, some signals detected by the other methods (p-value, GxG p-value, GxG BF) investigating SNPs or SNP-pairs marginally went undetected in some of the simulations where the causal SNPs had main effects.

• When causal SNPs with two-way interaction effects were considered, all methods considering GxG interactions (BMA, GxG p-value, GxG BF) yielded more satisfactory results than the marginal p-value.

• When causal models with three-way interactions were considered the BMA showed better performance than any of the alternatives.

The final aspect in the list above suggests that when higher-order interactions are present in data, taking them into account in the model may improve their detection. However, we further note that as the effect sizes got larger, even the simplest model, the marginal p-value, was able to identify most of the causal areas, even if the causal SNPs did not have main effects. The improved localization of the BMA method was most clearly seen when causal SNPs had main effects.

In general, the GxG Bayes factor seemed more competitive than the GxG p-value when compared with the BMA method. This may be partly explained by the fact that the GxG p-value handles only a single interaction model at a time, whereas the GxG BF considers an average of three different models.

Especially in the simulations with underlying three-way interactions, the model corresponding to the GxG p-value may be too limited to appropriately fit the complex interaction model, and consequently, a model with more parameters might perform better. On the other hand, increasing the complexity of the model would decrease the performance of the GxG p-value in the simpler simulation settings due to additional redundant parameters.

### Issues in the Bayesian model averaging

The bottleneck in terms of computational complexity in our approach is the enumeration of all locus pairs once and evaluating the posterior probabilities of the corresponding elementary models. After this has been done, the stochastic search increases only modestly the total time required. The *O*(*L*^2^) time complexity seems unavoidable for any method explicitly considering gene-gene interactions. Although the enumeration of all locus pairs is feasible with present day cluster computers even on the scale of GWA studies, a straightforward enhancement in terms of time intensity is to carry out a pre-screening of SNPs using e.g. a marginal p-value test with some liberal significance threshold, say, equal to 0.1. It is shown in [2] that such an approach leads to approximately equal power in identification of gene-gene interactions compared to the exhaustive enumeration of all gene pairs.

According to our experiences, the sensitivity with respect to the prior on the model structure may represent the primary obstacle to be appropriately handled when Bayesian model averaging type methods are applied to data sets with a very large number of SNPs. One solution provided by the Bayesian approach itself is that, instead of uniformly decreasing the value of the structure parameter *ξ *(penalty from adding another SNP to the model) as the number of SNPs in a data set increases, we could assign different *ξ_i _*parameters to different SNPs based on external knowledge about the particular loci [11]. For example, *ξ_i _*might be set equal to 0.01, 0.001 or 0.0001 depending on whether a SNP belongs to a gene whose function is expected to be related with the disease, any gene at all, or far away from any known gene, respectively. There does not seem to be a simple way for including continuous covariates in our model if one wishes to preserve the ability to analytically integrate out the model parameters, which constitutes the basis of efficient computation. On the other hand, including categorical covariates (such as sex, or age after some appropriate discretization) in our model is straightforward in principle, by treating them similarly as the observed genotypes. However, in practice the increase in the number of parameters may overwhelm the benefits. A possible solution might be to average over models such that the covariates are in turn either included or excluded. Finding an optimal way of doing this is subject to future research.

## Conclusions

We have considered the problem of identifying disease associated marker loci when several SNPs have a joint effect on the disease probabilities. We have introduced a novel Bayesian model averaging approach, whose advantages include explicit consideration of the GxG interactions, ability to describe higher-order interactions, and the ability to evaluate the marginal likelihood analytically enabling efficient computation. Our approximate model averaging algorithm makes it possible to include GxG interactions in the analysis even with large data sets. Furthermore, it would be fairly straightforward to modify the algorithm for learning with other model families than the one considered in this article, for example regression models commonly utilized in genetic association studies. Such a generalization would require that the model parameters can be integrated out either analytically or approximately using e.g. the Laplace approximation (see the supplementary material of [5]).

To conclude, our simulations confirm that an appropriate approach to initializing a GWA screening is to investigate marginally each SNP, either by p-values or corresponding Bayes factors (see, e.g. [11]), as this is the computationally most straightforward and fastest approach, and, in many cases, capable of finding the signals present in data. The relevance of the more complex modeling approaches including GxG interactions is that they may help to detect some causal SNPs which are not visible marginally. Thus, in our opinion, using different approaches side-by-side may provide a more detailed description of the data and aid in finding the missing heritability in complex diseases [23].

## Authors' contributions

PM had the main responsibility of the article. JC participated in the designing of the models, carried out some of the analyses and participated in the writing of the manuscript. Both authors have read and approved the final manuscript.
